# Editorial: Genome and transcriptome editing to understand and treat neuromuscular diseases

**DOI:** 10.3389/fgeed.2023.1176699

**Published:** 2023-03-14

**Authors:** Rika Maruyama, Alyson Fiorillo, Christopher Heier, Dongsheng Duan, Toshifumi Yokota

**Affiliations:** ^1^ Department of Medical Genetics, University of Alberta Faculty of Medicine and Dentistry, Edmonton, AB, Canada; ^2^ Center for Genetic Medicine Research, Children’s National Hospital, Washington, DC, United States; ^3^ Department of Molecular Microbiology and Immunology, School of Medicine, The University of Missouri, Columbia, MO, United States

**Keywords:** amyotrophic lateral sclerosis, 5′UTR, shRNAs, antisense oligonucleotides, GNE, microRNA, Duchenne muscular dystrophy, SOD1

Neuromuscular diseases such as Duchenne muscular dystrophy and facioscapulohumeral muscular dystrophy are debilitating conditions that affect millions of individuals worldwide. In recent years, there has been a growing interest in the use of genome and transcriptome editing techniques to understand and treat these diseases. This Research Topic brings together four articles that highlight the latest advances in this field. The first article “*A Single Transcript Knockdown-Replacement Strategy Employing 5′ UTR Secondary Structures to Precisely Titrate Rescue Protein Translation*” by Millette et al., presents a new strategy for precisely titrating rescue protein translation in cases of diseases caused by coding mutations such as amyotrophic lateral sclerosis (ALS). The authors developed a modular, single-transgene expression system that allows control over translation from high-expression, ubiquitous promoters. This system uses “attenuator” sequences in the 5’ UTR which predictably diminish the translation of the paired gene, providing wide general utility. The authors also demonstrate that this approach can be used to achieve a knockdown and rescue effect by pairing microRNA-adapted shRNAs alongside their respective replacement gene on a single transcript. They also showed that this approach can be used to replace the SOD1 gene in stable cell lines and demonstrate complete and predictable control over replacement of SOD1 by varying the strength of attenuators. This study highlights the potential utility of this approach in treating monogenic diseases caused by heterogeneous mutations. The second article “*Development of Therapeutic RNA Manipulation for Muscular Dystrophy*” by Saifullah et al., reviews the current state of therapeutic RNA manipulation for muscular dystrophies, specifically Duchenne muscular dystrophy (DMD). DMD is a severe monogenic disease caused by mutations in the DMD gene, leading to muscle degeneration and atrophy early in life and premature death. This article highlights the potential of oligonucleotide-based therapeutics, specifically exon skipping using antisense oligonucleotides (ASO), as a promising strategy for treating DMD. The authors summarize recent scientific and clinical progress of ASO and other novel RNA manipulations, and also mention the efficacy and limitations of these therapies to restore dystrophin. The article encourages the application of RNA-editing therapy to other neuromuscular disorders, given its great potential as a promising treatment for these diseases. The third article “*In vivo and in vitro genome editing to explore GNE functions*” by Ilouz et al. describes the use of genome editing techniques to study the functions of GNE, a gene associated with UDP-N-acetylglucosamine 2-epimerase/N-acetylmannosamine kinase (GNE) myopathy, a neuromuscular disorder characterized by slowly progressive distal and proximal muscle weakness. The authors used the CRISPR/Cas9 method for genome editing to add a tag to the endogenous GNE gene in mice, which allowed them to determine the spatiotemporal expression of the protein in the organism using well-established and reliable antibodies. They also generated a GNE knockout mouse muscle cell line to identify the events resulting from the total lack of the protein. By performing a multi-omics analysis of both cellular systems, the authors found that GNE is involved in cell cycle control and in the DNA damage/repair pathways. This study further illuminates the fundamental mechanisms of GNE action in normal muscle, which is expected to contribute to identifying which functions are disrupted in GNE myopathy, and the discovery of new biomarkers and therapeutic targets of this disease. The fourth and final article “*Gene Editing to Tackle Facioscapulohumeral Muscular Dystrophy*” by Mariot and Dumonceaux, describes the current state of research on CRISPR-based strategies for treating Facioscapulohumeral dystrophy (FSHD), a skeletal muscle disease caused by aberrant expression of the DUX4 gene in the muscle tissue. The authors discuss different approaches to editing the DUX4 gene at the DNA, RNA or protein levels, including epigenome editing, gene editing using TALEN, CRISPR/Cas9 or adenine base editing, and CRISPR-Cas9 genome editing for SMCHD1. They also discuss the challenges facing the development of these gene editing-based therapeutics. The authors point out that the recent development of CRISPR technology has opened up new possibilities for treatments for genetic muscular diseases and with this technology, a cure for these diseases can be considered for the first time. They also highlight that the lack of effective therapy for muscular dystrophies can be explained by the fact that more than 40 genes have been described to be involved in these diseases, resulting in a wide range of abnormalities and the large size of the mutated genes challenges classical gene replacement therapies.

Overall, these articles demonstrate the potential of genome and transcriptome editing techniques to improve the understanding and treatment of neuromuscular diseases. With the rapid pace of technological advancements in this field, it is hopeful that we will see even more exciting developments in the near future.

